# Energy-Efficient Wireless Sensor Networks for Precision Agriculture: A Review

**DOI:** 10.3390/s17081781

**Published:** 2017-08-03

**Authors:** Haider Mahmood Jawad, Rosdiadee Nordin, Sadik Kamel Gharghan, Aqeel Mahmood Jawad, Mahamod Ismail

**Affiliations:** 1 Department of Electrical, Electronic and Systems Engineering, Faculty of Engineering and Built Environment, Universiti Kebangsaan Malaysia, UKM Bangi, Selangor 43600, Malaysia; haider_mahmood2003@yahoo.com (H.M.J.); adee@ukm.edu.my (R.N.); Aqeel_mahmood_1986@yahoo.com (A.M.J.); mahamod@ukm.edu.my (M.I.); 2 Department of Computer Communication Engineering, Al-Rafidain University College, Filastin 10064, Baghdad, Iraq; 3 Department of Medical Instrumentation Techniques Engineering, Electrical Engineering Technical College, Middle Technical University (MTU), Al Doura 10022, Baghdad, Iraq

**Keywords:** energy-efficient, energy harvesting, precision agriculture, wireless communication technology, WSN

## Abstract

Wireless sensor networks (WSNs) can be used in agriculture to provide farmers with a large amount of information. Precision agriculture (PA) is a management strategy that employs information technology to improve quality and production. Utilizing wireless sensor technologies and management tools can lead to a highly effective, green agriculture. Based on PA management, the same routine to a crop regardless of site environments can be avoided. From several perspectives, field management can improve PA, including the provision of adequate nutrients for crops and the wastage of pesticides for the effective control of weeds, pests, and diseases. This review outlines the recent applications of WSNs in agriculture research as well as classifies and compares various wireless communication protocols, the taxonomy of energy-efficient and energy harvesting techniques for WSNs that can be used in agricultural monitoring systems, and comparison between early research works on agriculture-based WSNs. The challenges and limitations of WSNs in the agricultural domain are explored, and several power reduction and agricultural management techniques for long-term monitoring are highlighted. These approaches may also increase the number of opportunities for processing Internet of Things (IoT) data.

## 1. Introduction

Wireless sensor network (WSN) technologies have rapidly developed over the years. Ecological phenomena in a vast area can be monitored using pervasive devices called motes or sensor nodes. Battery-powered WSNs comprise several sensors, processors, and radio frequency (RF) modules. The sensor nodes or motes can communicate wirelessly through a communication link and forward their data to a base station or coordinator node by communicating with a gateway. The communication among sensor nodes depends on the merging of diverse sensors from simple (i.e., humidity, pressure, and temperature) to complex (i.e., localization, tracking, micro-radars, and images), thereby allowing WSNs to monitor a wide range of surroundings to obtain precise information from the field [1]. Accordingly, the sensing, storage, processing, and communication capabilities of sensor nodes have continuously increased [2]. 

WSNs have been used in different applications, such as military, agriculture, sports, medicine, and industry. Agriculture can be considered as one of the most favorable facilities for WSNs to improve food crop yields and minimize the burden of farmers [3]. Several projects have been introduced in the precision agriculture (PA) literature. PA aims to prevent the application of the same management routine to a crop regardless of site circumstances and to improve field management from several perspectives. For instance, PA can minimize the wastage of pesticides for the effective control of weeds, pests, and diseases as well as guarantee that crops receive an adequate amount of nutrients, thereby leading to a highly effective, green agriculture. Specifically, PA is a management strategy that employs information technology to improve agricultural quality and production. PA differs for traditional farming in the sense that this process accurately identifies variations and relates the spatial data to management activities. PA involves five stages, namely, (i) data collection, (ii) diagnosis, (iii) data analysis, (iv) precision field operation, and (v) evaluation [4]. 

WSNs are used as cost-effective processes to increase agricultural yield. Accordingly, WSNs have been used in different agricultural applications, such as for monitoring climate and using soil nutrient data to forecast the health of crops and the quality of agricultural products. Irrigation planning can be predicted using WSNs by observing weather conditions (such as temperature and humidity) and soil moisture. Other sensor nodes can be added to the existing WSN to improve the parameters of the agricultural monitoring system and to make the network scalable. However, some challenges have hindered the agricultural application of WSNs, such as determining optimum deployment schemes, measurement periods, routing protocols, energy efficiency, cost, communication range, scalability, and fault tolerance [5]. For example, the scattered deployment of sensor nodes with a long data-gathering period can help extend the network lifetime. However, some factors may challenge the selection of the distribution region. When the agricultural field faces many obstacles, the communication link may be weakened or lost as a result of signal attenuation. The sensor nodes in WSNs are supplied from the battery, thereby preventing connections to the main supply in the deployment location. Reducing power depletion and prolonging battery life are imperative for WSNs considering their limited battery power. Although the application of WSNs has increased constantly over the years, the manufacturing of batteries has not developed at the same rate [6]. Therefore, WSNs are mainly limited by their batteries [7]. Among the aforementioned challenges, this paper focuses on energy management and highlights energy-efficient and energy harvesting techniques that can be used in agriculture. 

To fulfill PA requirements, such as addressing the problems resulting from the long distance between farms and base stations, a mobile data connection service can be established by connecting unmanned aerial vehicles or drones with the base station [4]. Such connection allows the sensor nodes to relay their data to the base station within the wide area of the farm field. However, this solution is limited by the quality of service of WSNs. 

The main contributions of this paper are summarized as follows:

(i) The potential of using wireless communications protocols or technologies in agriculture was investigated, and the technology with the best power consumption and communication distance was identified.
(ii) The taxonomy of energy-efficient and energy harvesting techniques was examined to address the power consumption problems in agriculture and to identify those methods that are most suitable for solving these problems.
(iii) The existing solutions, applicability, and limitations of applying WSNs in agriculture were reviewed and compared.
(iv) Recent studies using WSN in PA applications based on Internet of Things (IoT) were surveyed and compared in terms of type of sensors and actuators, IoT end devices, IoT platforms, and IoT application layer.

## 2. Wireless Communication Technologies for Agriculture

This section presents the different wireless protocols and standards that are used in agriculture. These wireless technologies are also compared to identify the most convenient technology in terms of power consumption and communication range, where the two metrics are posed challenges in current solution of agriculture application [5,8]. IoT is a revolution for the future realm where everything that can utilize a connection will be connected. Cellular technologies are grown and developed to play a crucial role in the IoT realm [9]. Narrowband-IoT (NB-IoT) is a new IoT system constructed from current Long Term Evolution (LTE) functionalities. Subsequently, NB-IoT is possible to share the spectrum of LTE without coexistence problems and to utilize the same pieces of equipment, as well as it is possible to connect seamlessly into the LTE main network. This permits all network facilities such as security, tracking, policy, charging, and authentication to be totally supported [10]. The design goals of NB-IoT cover high coverage area, extended battery life (i.e., 10 years), high network size (52,000 devices/channel/cell), and low-cost devices [10,11]. However, in the near future, NB-IoT technologies, such as Long Range radio (LoRa) will take place in agricultural applications due to low power consumption and preferably used when the agricultural information are to be transmitted over long distances. 

### 2.1. ZigBee Wireless Protocol

ZigBee wireless protocol is considered one of the best candidate technologies for the agriculture and farming domains. Given its low duty cycle, ZigBee is considered appropriate for PA applications, such as irrigation supervision [12], water quality management, and fertilizer and pesticide control, all of which require a cyclic information update. Based on this technology, the sensor nodes in the agricultural field can communicate with the router or coordinator node over a long range (i.e., 100 m) when XBee Series 2 is used. ZigBee can also reduce the communication distance by up to 30 m for indoor conditions (i.e., greenhouses) [13]. The number of deployed sensor and router nodes will be increased to cover the entire field area under surveillance. Some studies have employed Zigbee for PA. For instance, ZigBee was used to investigate the effect of signal strength on node spacing, base station antenna height, and leaf density [14]. In [14], three experiments were conducted in palm orchards in to evaluate a signal propagation model based on the received signal strength indicator (RSSI) of the ZigBee wireless protocol. Through their experiments, the authors concluded that the wireless channel propagation model must be determined before deploying the sensor nodes in palm orchards to obtain robust signal strength characteristics. A path loss model in a mango greenhouse was investigated based on the ZigBee wireless protocol [15]. 

A cattle-localization-system-based ZigBee WSN was also applied in a grazing field [16]. Instead of requiring additional hardware, ZigBee relies on link quality indication (LQI) to measure distance. The ZigBee protocol achieves cattle localization at a low power consumption and minimum cost. The key parameters of greenhouse climate (i.e., humidity, temperature, CO_2_, and solar radiation) are considered in [17] to ensure the comfortable growth of plants with 22% energy savings and 33% water usage. ZigBee and Global System for Mobile communication/General Packet Radio Service (GSM/GPRS) technologies were used to monitor and control the climate condition of greenhouses [17]. The ZigBee wireless protocol has also been used to monitor animal behavior (e.g., walking, grazing, lying down, and standing) [18]. ZigBee was also adopted in [18] to overcome the high power consumption and unreliability issues in mobile ad hoc WSNs.

A WSN-based greenhouse that uses ZigBee star topology is merged with artificial intelligence (i.e., fuzzy logic controller) in [19]. The ZigBee wireless protocol is adopted to preserve energy by switching between active and sleep states. Thus, power consumption can be minimized, and the battery lifetime of sensor nodes can be extended. ZigBee is currently applied in smart beehives [20], orange orchards [21], dairy healthcare monitoring of cows in pastures and barns [22], automation in irrigation [23], greenhouse monitoring systems [24,25], and livestock monitoring [26]. As a universal standard for WSNs, the ZigBee protocol was used in several agricultural applications because of its low power consumption, low cost, self-forming characteristics, and suitable communication range. 

### 2.2. Bluetooth (BT) Wireless Protocol

The BT standard has been utilized to establish a communication link between movable and portable devices, such as laptops, over a short distance of up to 10 m. Given its pervasive nature and availability in most mobile devices, BT has been employed to satisfy multi-level agricultural requirements [5]. Weather information, soil moisture, sprinkler position, and temperature are monitored remotely using Global Positioning System (GPS) and BT technologies. The proposed system in [27] was developed for irrigation application to increase field productivity and conserve water. The irrigation application proposed in [28] collects field information in real time via the BT wireless communication protocol. Several software and hardware were developed in [29] to monitor the relative humidity and temperature in greenhouses based on BT. The BT module was employed in integrated control method [30] to control the irrigation system in greenhouses based on soil and weather information, and this technology improved the leaf number, height, dry weight, and fresh weight of red and romaine lettuce in greenhouses. Result of estimation for water usage and electricity also improved by 90% based on integrated control method using BT technology relative to the traditional method (i.e., timer control strategy). Given its low energy consumption, wide availability, and ease of use for farmers, smartphone-based BT has been employed in different agricultural applications [31,32,33,34,35], such as controlling irrigation systems, monitoring soil and weather conditions, and controlling the use of fertilizers and pesticides. 

### 2.3. WiFi Wireless Protocol

WiFi is currently the most extensively utilized wireless technology available in portable devices, including tablets, smartphones, laptops, and desktops. WiFi has a suitable communication distance of about 20 m and 100 m in indoor and outdoor environments, respectively. In PA applications, WiFi extends diverse architectures by connecting several types of devices via an ad hoc network. WiFi and 3G wireless technologies were employed in [36] to estimate the agricultural applications of mobile phones. Remote access and short message services have also been used for controlling and monitoring protected crops. In [37], agricultural data, such as soil temperature, soil moisture, weather temperature and humidity, sunlight intensity, and CO_2_, were stored in a gateway before they were transmitted to the server computer over a WiFi network. A Wi-Fi (IEEE 802.11g)-based smart WSN is proposed for agricultural monitoring in [38]. The proposed system consists of three nodes: sensor, router, and server. The climate conditions of the greenhouse or agricultural field, such as humidity, temperature, air pressure, light, water level, and soil moisture, are monitored. The said work attempts to lower cost, minimize wiring connections, and enhance the mobility and flexibility of the sensing points in WSN. However, the energy consumption of the proposed system is extremely high at 42.17 J/h.

Given that WiFi requires much power [5], long communication time, and huge data payload [26]. WiFi technology is not preferable for agricultural applications in spite of the fact that a Wi-Fi server prevents data losses by adopting data redundancy techniques. In addition, WiFi cannot be employed for multi-hope applications and influenced by number of users and the signal intensity; thereby, it is inappropriate for agriculture WSNs. Moreover, the WiFi nodes listen all the time, so power consumption will increase [39]. 

### 2.4. GPRS/3G/4G Technology

General Packet Radio Service (GPRS) is a packet data service for GSM-based cellular phones. GPRS frequently experiences variable delays and throughputs, and such technology depends on the volume of consumers that share the same communication channels and resources. Gutiérrez et al. [40] used the GPRS module and WSN to develop an automatic crop irrigation system based on the information collected by temperature and soil moisture sensors installed at the root zone of plants, and considered this system a cost-effective and practical solution for improving water quality in PA. A drip irrigation process was evaluated in [41] by measuring soil moisture. A prototype system was also developed based on a data management server and a WSN-GPRS gateway. WSN-GPRS gateway acts as a bridge between WSN and GPRS where the data from the WSN are transferred to a data management center. Navarro-Hellín et al. [42] equipped various wireless nodes with GPRS to measure and transmit soil, plant, and atmosphere information. The wireless nodes have unlimited autonomy because of their independent nature and use of solar energy. Different sensors may transmit information to a remote location via a GPRS network for further analysis by using tablets, mobile phones, or computers. All agriculture sensors are interfaced to the sensor board to obtain agricultural information. Such information is transmitted to the remote server for further analysis through the GPRS-board, which depends on a GSM/GPRS mobile network.

### 2.5. Long Range Radio (LoRa) Protocol 

LoRa is introduced by the LoRa Alliance as a protocol stack for low power and wide area Internet of Things (IoT) communication technologies that are associated with indoor transmission [43]. The basic network architecture of a LoRaWAN consists of LoRa end devices, LoRa gateways, and a LoRa network server. The LoRa end device communicates with gateways that employ LoRa with LoRaWAN. LoRa gateways pass raw LoRaWAN packets from the end devices to a LoRa network server with a high throughput based on the backhaul interface, which is typically 3G or Ethernet. Accordingly, LoRa gateways act as a bidirectional communication or protocol adapter with the LoRa network server. In this case, the LoRa network server takes charge of decoding the data packets transmitted by the LoRa devices and creating the frames that would be directed back to the devices. LoRa offers a bidirectional solution that matches machine-to-machine (M2M) WiFi or cellular technologies. LoRa presents a cost-effective method for linking batteries or mobile devices to the network or to end devices. The LoRa wireless protocol was used in [44] to monitor bee colonies in rural areas and to ensure a communication between the bee node and the local server that is located in a remote location. The soil moisture and temperature, air temperature and humidity, and light intensity inside greenhouses were also monitored using different sensors, microcontrollers, and the LoRa wireless protocol [8]. The LoRa gateway collects data from LoRa nodes to construct the topology of a star network, and may communicate with a cloud server over a long communication range and with high scalability.

LoRa was recently utilized in several agriculture research projects developed by the Libelium Company (Zaragoza, Spain) [45], such as improving kiwi production based on a smart irrigation system (Italy), monitoring of green areas by using a smart garden system (Spain), improving fertilization methods of corn yields (Italy), improving production of banana yields (Colombia), increasing tobacco crops by controlling the climate condition (Italy), saving water through a smart irrigation system (Barcelona), monitoring of vineyard crops (Spain), and monitoring of diseases that affect vineyards (Switzerland). 

### 2.6. SigFox Protocol

SigFox is an ultra-narrowband wireless cellular network [43] with low data rate applications, thereby making this technology appropriate for IoT and machine-type communications systems. SigFox has been used in different applications, including telephone, security, mobile, broadband, and television. The SigFox network was used in [46,47] to construct a geolocation system that localizes animals in mountain pastures throughout the summer. A system was proposed in [46] to help farmers locate their cattle and enhance their production. The importance of power consumption analyses was highlighted in [47], especially when the cattle are located in high mountainous areas.

### 2.7. Performance Comparison of Wireless Communication Protocols

Table 1 compares the aforementioned wireless communication protocols or technologies relative to different parameters, including power consumption, communication range, data rate, cost, system complexity, and other parameters. The challenges in agricultural applications may be developed from the selection of the deployment range. For instance, the transmitted signal by the sensor node is attenuated when the agriculture field is separated by obstacles. Power consumption is considered as another limitation in the WSN design in agricultural applications [5]. The ZigBee wireless protocol was designed to run with a suitable communication range and low power consumption. LoRa and SigFox are considered to work with low power consumption and long radio range. Accordingly, the power consumption and communication distance of the above technologies have been assessed as follows. 

Zigbee and Bluetooth low energy (BLE) are designed for battery-powered devices. These technologies conserve power through low duty cycling and enter sleep mode to extend the battery lifetime. Classic BT, WiFi, GPRS, LoRa, and SigFox have a higher power consumption than ZigBee. Although ZigBee has a shorter communication range than LoRa, SigFox, and GPRS, this range may be extended with a router node to overcome the node deployment limitations in agricultural applications. BLE outperforms ZigBee in terms of power consumption, but this wireless protocol is limited in agricultural applications because of its short communication distance of 10 m, as reported in [48]. ZigBee also has a higher network elasticity than BT, thereby permitting various topologies. Given the large number of nodes (more than 65,000) in the ZigBee network, the ZigBee technology can be used to cover a wide agricultural. Accordingly, the ZigBee wireless protocol has been adopted in many agriculture studies [5,49,50,51,52,53]. One of these studies [5] prefers to utilize ZigBee in farming domains and agricultural applications owing to its energy-efficient, reliable, and low-cost wireless protocol.

The LoRa wireless protocol covers a large communication area with low power consumption. Therefore, it can be suitably deployed in vast agricultural fields. The network size of the LoRa protocol is limited by its duty cycle, where a 1% duty cycle can lead to a maximum communication time of 36 s per hour for each end device [54]. In addition, LoRa gateways have a limited message capacity of less than 100 messages per minute because of the unplanned deployment of LoRa gateways in urban areas, thereby interfering with low power wireless area networks, such as SigFox. Therefore, increasing the number of gateways can challenge the infrastructure of the LoRa network [54]. Moreover, the LoRa technology is complex and has sizable hardware implementations [55]. 

SigFox has an extremely low data rate (100 bps) and slow transmission time (10 s for 10 bytes) compared with ZigBee and LoRa [56]. SigFox supports star topology supervised by a centralized sever that manages connectivity between devices and the base stations, whereas the LoRa supports wide area network based on star-to-star topology creating a bridge of LoRa gateways. Given that data rate and transmission time are both critical issue in any communication system, SigFox cannot be widely used for agricultural applications. Moreover, this technology does not support the collision avoidance scheme, thereby affecting the network because several M2M devices may be operated in the same area. Moreover, SigFox has a limited reliability because of its one-way communication (without an acknowledgment signal) and low received power. However, both the LoRa and SigFox infrastructures are still in progress. Ali et al. further discuss the limitations of these protocols [56]. 

**Figure 1:**
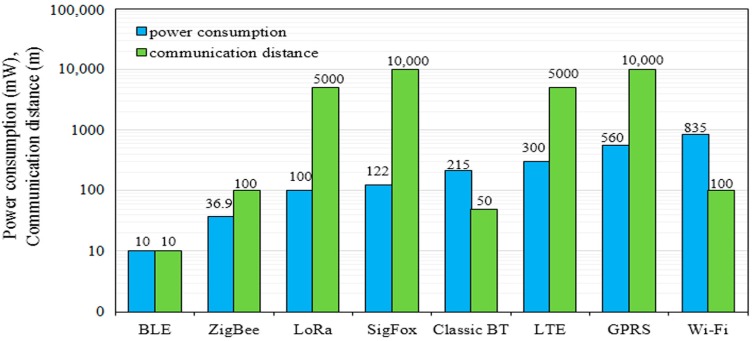
Different wireless technologies in terms of power consumption and communication distance.

From the above discussion and the comparison results presented in Table 1 and Figure 1 and as will be shown in Table 2, Table 3, Table 4, Table 5, Table 6, Table 7 and Table 8 the ZigBee and LoRa wireless protocols have been identified as the most suitable wireless protocols for agricultural applications because of their low power consumption, communication range (acceptable for ZigBee and long for LoRa), small size, easy network implementation, simplicity, low latency (for ZigBee), scalability, and network size.

PA using sensors, actuator, processor, wireless transceiver, and other information technologies, potentially permits manufacturers to computerize site-specific management for automated agriculture. In terms of precision, the considered wireless protocols for agricultural applications have improved the agriculture domain. Precision irrigation is frequently regarded as optimizing irrigation accuracy due to its ability to control the optimum amount of water and irrigation time. The water savings of 90% (based on ZigBee), 33% (based on ZigBee), 50% (based on Bluetooth), and 90% (based on Bluetooth) were achieved for precision irrigation systems relative to traditional irrigation systems in research [17,28,30,40], respectively. Animal behavior, such as laying down, standing, grazing, walking, and other modes, was monitored in [18] using ZigBee wireless protocol. The authors found that laying down and grazing could be improved by 83.5% compared with the findings from similar studies. In another study that has adopted a proposed algorithm that depended on the ZigBee wireless protocol [20], an accuracy rate of 95.4% was expected for the proposed algorithm to monitor the honey bee and the agricultural and environmental aspects. In [23], the automation of the irrigation system based on ZigBee saved the system cost between 1.24% and 6.72% relative to the entire cost of the water user associations, with energy savings of 2.05 to 8.21% and water savings of 0.71 to 6.46%. The proposed model in [35] utilized a smartphone platform (based on Bluetooth and GSM technologies) and permitted three types of irrigation systems. The efficiency factors were improved by 90% for subsurface drip type and 85% and 75% for high- and low-pressure overhead sprinkler types, respectively, to compute for drift and evaporation before the water droplets approached the soil. 3G technology was adopted by Libelium based on Waspmote wireless sensor devices to monitor the vineyards in Northwest Spain [57]. Phytosanitary treatments, such as fungicides and fertilizers, were minimized by 20% and growing yields were enhanced by 15% based on PA. In [8], LoRa wireless protocol was considered to monitor the agriculture environments with 90% power efficiency. The cost of pumping and water for precision irrigation system in green areas was reduced by 30% based on SigFox wireless protocol [58].

The preceding survey for different agricultural applications revealed that PA can be achieved in terms of water savings, animal behavior, accuracy, power efficiency, and reducing system cost for various wireless protocols. Consequently, agriculture can be improved based on agricultural automation systems compared with traditional agriculture systems.

## 3. Agriculture-Based Energy-Efficient Schemes in Literature

**Figure 2:**
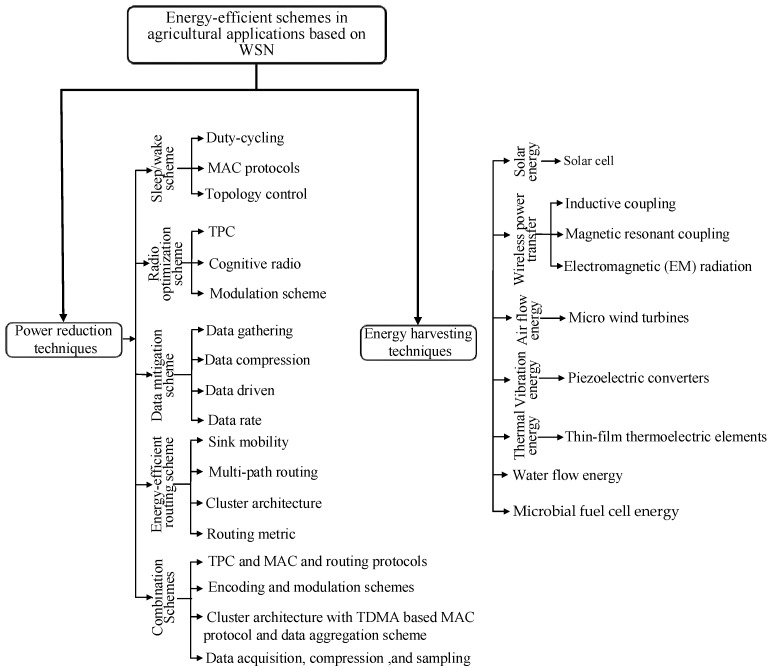
Energy-efficient schemes in agriculture based on wireless sensor networks (WSNs).

The solution to the power consumption problem in the agriculture domain using WSNs has been highlighted in several studies. Previous studies focused on reducing the power consumption of sensor nodes in WSNs by proposing energy-efficient schemes. This study reviews the existing energy-efficient schemes in WSNs as shown in Figure 2. This review classifies the energy-efficient schemes in agriculture application into two categories, namely, power reduction and energy-harvesting techniques. These techniques can also be classified into subcategories to explore the power consumption problem of agriculture WSNs. Most of these techniques can be employed in agricultural applications, which are highlighted in the following subsections.

### 3.1. Agriculture-Based Power Reduction Techniques

WSNs contain numerous sensor nodes, which are utilized to measure ecological phenomena in real time and transmit information back to the master node through a wireless module. Given the absence of wire connections, sensor nodes are appropriate for different applications under strict environments. PA is one of the important applications relying on WSNs [67]. A sensor node is usually equipped with rechargeable batteries, which have limited capacity and pose a challenge to long-term application [68]. These batteries supply power to the sensor nodes by providing the necessary current to maintain each part of the sensor nodes working properly. The total power consumption of a sensor node is the sum of each element in the node (i.e., sensor, microcontroller unit, and radio module), and each component may operate at different energy states. Therefore, the lifespan of a sensor node is the time consumed to exhaust its batteries under a sustainable operation threshold.

Numerous scholars have developed power reduction techniques to provide sensor nodes an infinite lifespan. This section reviews several power reduction techniques of WSNs that can be used in agricultural applications. The purposes, wireless protocols, findings, advantages, and limitations of each technique are also discussed. 

#### 3.1.1. Sleep/Wake Strategy

Wireless transceivers consume a significant amount of power relative to processors. The energy of the wireless nodes is mostly dissipated during the reception and transmission processes. The radio of sensor nodes can enter sleep mode via the *sleep/wake* strategy to reduce the power consumption of the RF components of WSNs. No data communication occurs during the sleep mode. The sensor nodes wake up to collect and transmit data within a certain period and then return to sleep mode to conserve energy. In agricultural applications, the *sleep/wake* strategy is implemented via (i) duty cycling, (ii) medium-access control (MAC) protocols, and (iii) topology control.

*Duty cycle* is proposed in several studies to lower the power consumption for different agricultural applications based on WSN. Zhang et al. [69] proposed a WSN-based system to monitor soil moisture and analyzed the temporal and spatial variability of soil moisture for variable irrigation. To reduce the power consumption of the soil sensor nodes, the soil moisture sensors were always in sleep mode and activated only when data were to be collected. Million et al. [70] designed a WSN for PA in Malawi and called it WiPAM. This system was intended to automate irrigation and implement an irrigation management system (IMS). The authors investigated soil moisture variability. The sensor nodes were homogeneous, and each was composed of a ZigBee end device (ZED) and a ZigBee coordinator (ZC). In the star topology, three sensors nodes were configured as ZED (in field-nodes) and one as a gateway node. Another node was configured as ZC for aggregating data and actuating the irrigation valves. The power source to sustain the operation of the network was solar power. This approach was complemented by an energy-efficient management approach. In other words, sensor nodes went to sleep when idle and woke up to transmit data when required. The irrigation valves opened or closed depending on the values stored in the coordinator node. Ouadjaout et al. [71] presented an energy-efficient WSN for soil moisture monitoring in an irrigation system. The new platform, DZ50, consists of an RFM12b transceiver and an ATmega328P microcontroller. This platform consumes very low power, and much energy can be saved in sleep mode, in which the transceiver goes inactive (sleep mode) for long periods. Comparison showed that the performance of DZ50 is better than that of TelosB and MicaZ. Specifically, the battery lifetime of DZ50 is seven times that of TelosB and MicaZ based on a 10 s sampling period. Monitoring of environmental conditions, such as wind direction, wind speed, humidity, temperature, conductivity, rain level, pH, and water level, in crop fields was presented in [72]. A solar cell was used to power several sensor nodes deployed at a distance of 500 m to 5 km. WSNs based on IEEE 802.15.4 (C1120 transceiver) and GPRS/3G were adopted to transmit the rural environments to the base station. Energy was saved through low duty cycling (3.3%) for the RF transceiver; the saved energy is equivalent to 30 s of wake up time every 15 min. In addition, a 2-watt solar cell was used to supply power to the overall system, whose total average power consumption was 207 mW. 

*MAC protocols* were also proposed in previous research on agriculture WSNs [39,73,74,75,76]. Sudha et al. [39] proposed an energy-efficient TDMA-based algorithm for wireless sensor communications in an automatic irrigation system. Their experiment involved a header node that collected data directly from all the nodes in the field and passed these data to the base station (BS). This network arrangement was a star single-hop topology. These techniques attain power savings of 10% with a high communication distance of 1 km between the sensor node and the base station. However, when this distance increases, the power consumption also increases. López et al. [75] proposed a WSN for monitoring horticultural yields, which are disseminated among small plots scattered at a communication distance of 10 km. The air temperature and soil moisture are collected during a 10-week period. The authors adopted the MAC protocol and star topology to conserve the power consumption of the sensor nodes that used the IEEE 802.15.4 standard based on the CC2420 transceiver. The lifetimes of the soil moisture and air temperature sensor nodes are 1024 days and 222 days, respectively, utilizing a battery that delivers 2700 mAh. 

The *topology protocol* scheme can be used in agricultural monitoring WSNs to modify the network topology according to the application requirements, thereby reducing the number of active nodes in a network. Some nodes in the network can enter sleep mode to prolong the network lifetime when they are not sensing the same area of interest. Aneeth and Jayabarathi [77] implemented Ad hoc On-Demand Distance Vector (AODV) and Dynamic Source Routing (DSR) protocols over a different topology of ZigBee WSN (i.e., cluster topology, random topology, star topology with gateway based on two cases; inside and outside) in simulation environments by using the OPNET software (Riverbed, San Francisco, CA, USA). Twenty soil moisture sensor nodes and one gateway node were distributed in a farmland with a dimension of 100 × 100 m^2^. Each node sends information about the soil moisture to the gateway node through the ZigBee wireless protocol. The gateway node passes the information to the controlling system to control the irrigation valve system. Their study showed that the star topology using DSR protocol reduces the power consumption of the WSN because it has less routing overhead compared with AODV protocol. Table 2 shows the comparison of power reduction technique based on *sleep/wake* schemes for previous research in agricultural applications. 

#### 3.1.2. Radio Optimization 

Previous studies show that the power is mostly dissipated in the RF components of WSNs than in data processing units, such as microcontrollers and microprocessors [80]. A number of scholars have applied various radio optimization schemes or techniques, including (i) transmission power control (TPC), (ii) modulation scheme, and (iii) cognitive radio, to reduce the power consumption of the RF components of agriculture sensor nodes. In the *TPC scheme*, the sensor nodes modify the transmitted power to save energy, stimulate avoiding interference, and establish a communication link [81]. TPC can be used in the agricultural field [82,83], where the RF transmitted power of the sensor nodes can be modified to reduce their power consumption based on the measured distance between the sink node and the sensor nodes. Sahota et al. [82] have investigated the application of TPC in reducing the power consumption of the sensor nodes in precision agriculture-based WSNs. Several power levels and different receiver sensitivity levels are considered to perform the said process. The network layers and MAC protocol in a WSN (based on CC1110) for the agriculture domain are adopted to further lower the power consumption of the sensor node. Results show that the power saving that employs multiple modes of transmitted power can be improved by approximately 10% relative to the traditional mode. By contrast, Kamarudin et al. [83] prolonged the lifetime of the sensor node by more than 8.5% by modifying the transmitted power of the CC2420 communication device.

A *cognitive radio* is an intelligent wireless communication network [84] in which the wireless communication channel in the spectrum band can be selected efficiently [85]. The transmission metrics (e.g., transmitted power, carrier frequency, and modulation scheme) can be adjusted accordingly. A cognitive radio requires more energy than conventional devices do because it includes sophisticated and complex functions [86]. Therefore, an energy-efficient cognitive radio network poses challenges, especially with regard to the use of battery energy. In wireless communication, the electromagnetic spectrum is fully occupied, with several bands being partially used or unused. This situation supports the utilization of a cognitive radio, in which spectrum holes can be utilized.

Sabri et al. [19] fused fuzzy logic control (FLC) with a ZigBee wireless sensor actor network (WSAN), which can sense the surroundings and respond in ideal performance without interference from agricultural crops. FLC assists the specialists in managing complex systems more compared with traditional control methods, which are ineffective in terms of flexibility, energy, productivity, and labor interference. Two significant greenhouse environment parameters (i.e., humidity and temperature) are monitored during daytime and nighttime. The results show that the intelligent technique-based FLC can significantly improve the WSAN lifespan. Therefore, the WSAN can work for 14 years with a 210 mAh battery power based on the FLC merged with WSAN.

The power consumption of networks can be directly affected by the *modulation scheme* [87]. The modulation parameters can be adjusted to their optimal values to preserve the minimum power consumption of radio modules. Anane et al. [88] investigated the Frequency-shift keying (FSK) and Minimum-shift keying (MSK) modulation strategies to minimize the total power consumption of the sensor node required to transmit given data packets. Through their extensive study, the authors concluded that MSK outperforms FSK in terms of power consumption. However, several modulation schemes cannot be used in the ZigBee wireless protocol that operates at 2.4 GHz because this protocol supports a single modulation scheme. Specifically, the ZigBee wireless protocol works with offset quadrature phase-shift keying (OQPSK) [89]. Table 3 shows the comparison of power reduction technique based on radio optimization schemes for previous research in agricultural applications. 

#### 3.1.3. Data Mitigation

*Data mitigation* presents another power consumption solution for agriculture WSNs to reduce the amount of data transmitted from source nodes to destination nodes. Data mitigation can be achieved via (i) data gathering, (ii) data comparison, (iii) data rate, and (iv) data-driven techniques.

*Data gathering* can extend the lifetime of sensor nodes in WSNs by minimizing redundant data transmission. In different applications of agriculture WSNs including environment observation, the sensed data of the neighboring nodes may spatially be associated. In this case, data combination presents an important method for reducing the amount of redundant data, reducing the number of transmissions, and minimizing the power consumption of the nodes in the network [90]. Data gathering also reduces latency by minimizing data traffic and improving the delay between the sink and sensor nodes; after reducing the number of communications, this approach can improve the exploitation of bandwidth [91]. Data merging can be accomplished along a route starting from sensor nodes to sink nodes. The nodes in a route can rebroadcast only the maximum, minimum, or average amount of received data. The design of an energy-efficient WSN-based data-gathering scheme poses a significant challenge because this scheme must ensure a trade-off among power consumption, latency, security, data accuracy, and fault tolerance. 

Gathering data in agricultural applications, including humidity, weather, temperature, and soil moisture, can minimize the power consumption of environment sensor nodes by spatially correlating the field and redundant data of the sensor nodes when the temperature does not change significantly [1], especially during nighttime. For example, if the microcontroller of the temperature sensor node measures the same temperature value for extended hours, then the sensor node sends the temperature value every four hours to reduce power consumption. Merging the temperature and humidity sensors into a single sensor may also reduce the power consumption, size, and system complexity. Azaza et al. [17] integrated climate condition parameters (i.e., temperature, CO_2_, humidity, and illuminance sensors) inside a greenhouse and observed a comfortable growth of plants while saving water resources and energy. The greenhouse was equipped with environment control devices, such as ventilation system, fogging system for humidification, and electrical heaters. A smart system based on fuzzy logic control correlated humidity and temperature measurements is implemented to improve the fogging system operation. In that way, the power consumption of the fogging system can be significantly reduced. In addition, the system was improved by a wireless data observing platform (STM 32/F4 microcontroller and Virtex V FPGA board) for logging and data routing, which provides data access in real time. The suggested control system based on fuzzy logic control was experimentally implemented and validated. The results revealed that the total energy and water usage decreased by 22% and 33%, respectively.

Musaazi et al. [92] introduced a data caching algorithm (DCAL) that optimizes the sleep/wake up periods of ZigBee WSNs to achieve low energy consumption and latency in PA application. In WSNs, data are collected from the source nodes and routed to the sink node; from the sink node, the data are sent to the Internet. Reducing the transmission of duplicated sensed data reduces the power consumption. Energy conservation is paramount during the communication phase to prolong the lifetime of sensor nodes. The main method utilized to conserve energy involves switching off the nodes’ transceiver when they are not transmitting nor receiving packets. Additionally, the data-gathering method for agriculture WSNs has been investigated in [93]. The proposed system includes ZigBee wireless sensor nodes, customized farming personal digital assistants (PDAs), and a host PC. Each sensor node contains four sensors to identify five agricultural field parameters: soil temperature, soil moisture, environmental humidity, soil electrical conductivity, and environmental temperature. The PDA combines the ZigBee coordinator node, GPRS module, and GPS module. The ZigBee WSN gathers farming data and obtains GPS information for each sensor node. It then passes the data to the host PC through GPRS. The experimental results show that the battery power of the sensor nodes can be saved based on the data-gathering scheme. 

*Data compression* is a useful method for deploying sensor nodes with restricted energy resources. Information encoding can be performed in sensor nodes, whereas information decoding can be performed in sink nodes. Using compression techniques to reduce the size of information that the sink nodes transmit over wireless channels may also reduce the power consumption and extend the battery life of the sensor nodes. One study has applied data compression approaches to reduce the power consumption for auto-irrigation system which based on CC1000 RF module [41]. Based on this method, the battery of the sensor node can be extended to 359 days.

The *data-driven* power reduction technique aims to reduce the amount of sampled data by conserving the sensing accuracy to an appropriate level for a specified application [94]. Thereby, the amount of data to be transmitted to the sink nodes can be minimized. Therefore, this technique can reduce the power consumption of agriculture WSNs and extend their battery life. A data-driven algorithm is considered by Lerdsuwan and Phunchongharn [95] to solve the energy consumption problem of the sensor node in PA with low complexity and maintain the transmission rate. Their experiment reveals that their proposed algorithm has higher energy efficiency than the ESPIN and SPIN algorithms in [96,97] by 36.84% and 81.53%, respectively.

The *data rate* of RF transceivers can be adjusted in the physical layer of a wireless protocol to reduce the power consumption of the sensor node. Davis et al. [98] investigated the low battery condition of a sensor node to attain prolonged battery life for separate wireless sensor nodes that can preserve sampling suitability and reliability in communicating accurate data. Different sensors, such as those for soil moisture, sap flow, and soil water, were examined to study the effect of sensing debility due to low battery voltage. The comparison of the data sampling rate of these sensors shows that sap flow measurement is the best option for agricultural WSN. This finding is attributed to the low error obtained when the sampling interval increased to 15 min while maintaining measurement accuracy. Table 4 shows the comparison of power reduction technique based on *data mitigation* schemes for previous research in agricultural applications. 

#### 3.1.4. Routing Protocol

*Routing protocol* introduces another power reduction scheme for agriculture WSNs to minimize the path between sensor nodes and sink node, thereby the power consumption of the WSN is reduced. Routing protocol can be performed via (i) sink mobility, (ii) multi-path routing, (iii) cluster architecture, and (iv) routing metric. 

*Sink mobility* was adopted in previous research [99,100]. Mathur et al. [99] proposed a novel mechanism that adopts unmanned aerial vehicles (UAV) to gather data from forest regions instead of classical sensing from nodes deployed in WSN. The proposed method enables the UAV to collect data from WSN in harsh terrain and transmit it to the base station situated far from the sensing area. Therefore, the multi-hop transmission between cluster heads can be completely avoided, and the communication range can be extended.

*Multi-path routing* has also been suggested by other scholars for agriculture WSNs [67,68,101]. Recently, Nikolidakis et al. [67] proposed an agricultural field weather monitoring system based on the ZigBee wireless protocol. An advance energy-efficient routing protocol was implemented for WSN to automate irrigation management. The transmitter could modify its power based on the distance between the sensor node and base station to save energy. The results revealed that network lifetime increased to 1825 min. Hedley et al. [101] developed a WSN soil moisture mapping and monitoring method to provide information for irrigation scheduling. To conserve power, the sensor nodes relayed data to the BS every 15 min through the greatest energy-efficient route. 

*Cluster head* method is considered by Ndzi et al. [102] to extend the ZigBee WSN lifetime for agricultural application. In addition, the quality of the crop yields is improved and their cost is minimized. The research presented WSN coverage measurements in a mixed crop farmland. An adaptive energy consumption model for each sensor node was proposed and used to compute the energy consumption in the network. A deployment model with a cluster head and two antennas was also proposed and simulated to alleviate short network lifetime due to the vegetation attenuation of signals. This network deployment model extended the lifetime of the network by a factor of more than 20 compared with a deployment where cluster heads are not used. Nikolidakis et al. [103] proposed a new routing protocol for WSN that can reduce the power consumption of sensor nodes through balanced clustering. This protocol was called “equalized cluster head election routing protocol” (ECHERP). ECHERP was implemented in Java language for 500 homogeneous nodes distributed in a 100 × 100 m^2^ land. The simulation results showed that the proposed protocol outperforms existing protocols in terms of power consumption. A potential application of this protocol is PA.

Another type of routing protocol is called *routing metric*. Chen et al. [104] used two routing metrics, namely, remained energy and expected transmission count, to compute the optimal routing path to the sink node that ensures low power consumption. “A scalable context-aware objective function (SCAOF)” is proposed by combining robustness-aware, energy-aware, resource-aware, and reliability-aware contexts that render to the compound routing metrics scheme. The experiment is conducted in a garden based on a testbed, which includes a uIPv6 protocol stack and contiki OS. The obtained results prove that SCAOF can convey the desired advantages on network lifespan prolonging, as well as high efficiency and reliability in different hardware testbeds and simulation scenarios. Table 5 shows the comparison of power reduction technique based on routing protocol schemes for previous research in agricultural applications. 

With these power reduction techniques, we compared the power saving or battery lifetime, communication distance, and limitations of WSNs in different agricultural applications in the work of several scholars (Table 2, Table 3, Table 4, Table 5 and Table 6). The tables show that most previous studies used ZigBee wireless protocol for different agricultural applications. The use of ZigBee supports our assumption that this wireless protocol is suitable for agricultural monitoring systems as discussed in Section 2. A combination of two or more power reduction schemes was also highlighted by several scholars [106,107,108] to further lower the power consumption of the WSN. Table 6 shows the comparison of power reduction technique based on a combination of power reduction schemes for previous research in agricultural applications. Considering that sensor nodes are power-hungry and battery-limited, one or more of the power reduction schemes can be integrated with energy harvesting techniques to prolong the operation life of sensor nodes significantly. The classification of energy harvesting techniques is explained in the following section. 

### 3.2. Agriculture-Based Energy-Harvesting Techniques 

A major limitation of sensor nodes is their limited battery capacity. Several energy-efficient schemes have been introduced in previous research to solve the power consumption problem of sensor nodes. An alternative method that has been utilized to address the problem of limited node lifespan is the use of energy-harvesting techniques. Numerous techniques have been developed to allow sensor nodes to harvest different types of energy, such as solar, wireless power transfer (WPT), mechanical vibration, kinetic, and wind energy, from different environments [110] (Figure 2). Compared with traditional sensor nodes, rechargeable sensor nodes can work continuously for a long lifetime. Ambient energy can be converted to electrical energy and used directly to supply the sensor nodes or can be stored and used later on. In agricultural applications, energy harvesting can be used to prolong the lifetime of sensor nodes. Energy-harvesting techniques for previous research works employed in precision agriculture are shown in Table 7. The said table lists the categories of energy-harvesting techniques, types of wireless protocols, and output energy/power of energy-harvesting techniques. In addition, the form of agricultural applications, and limitations of each article are also highlighted.

Energy-harvesting mechanisms can be implemented with batteries for the sensor nodes. For example, a sensor node using solar energy to charge its batteries can work extensively during daytime when adequate sunlight is available. Meanwhile, the sensor node can use its batteries through power reduction techniques, such as sleep mode (i.e., duty cycle), at night when sunlight is unavailable to save energy. In addition, the sensor node can enter restricted sleep periods (i.e., low duty cycle) and reduce transmission power when its batteries possess low residual energy [111]. Furthermore, adopting a maximum power point tracking system is a reliable battery charging technique for long-term operation. The charge–discharge cycle of the battery is minimized [112].

#### 3.2.1. Solar Energy 

*Solar energy* based on photovoltaic system and methods can be used in agricultural applications based on WSNs [113]. Therefore, solar cells provide a good solution [114] to ensure the survivability of the agriculture monitoring system. Solar cell energy has been used by several studies to provide long-term energy to sensor nodes in agriculture applications. 

Gutiérrez et al. [40] developed an irrigation system based on the ZigBee wireless protocol. The system was developed to improve water usage for agricultural crops. Temperature and soil moisture sensors in WSN were placed in the root of the plants to send sensor information to a web application via a gateway. The proposed algorithm controls the water quantity based on threshold values of soil moisture and temperature that are programmed in a microcontroller-based gateway. The WSN is powered by solar cell panels and rechargeable batteries. Water savings of up to 90% were achieved relative to the conventional irrigation method. Considering that the system is cost effective and autonomous, it can be utilized in geographically isolated regions where water sources are limited.

Zou et al. [68] improved the battery lifetime of a WSN based on harvested energy (i.e., solar cell) utilizing shadow detection. With this strategy, sensor nodes can accordingly modify their scheduling to optimize their power production and remaining battery levels. Furthermore, data transmission is optimized through routing and clustering mechanisms. A Bayesian network was employed to provide warning reports of bottlenecks along the path. The system was deployed in real time based on the Texas Instruments CC2530 platform that includes the ZigBee wireless protocol. The battery (lithium type with a storage capacity of 2500 mAh) of the sensor node was charged from the solar cell panel when the harvested power was adequate. The experimental results showed that these techniques allow network activities to occur in a continuous and effective manner. 

In [115], a micro-irrigation system that relies on a small solar-powered cell was developed. The energy input, energy output, greenhouse gas productions, and solar cell installation for rural areas were investigated with the proposed co-located system. The researchers compared their system with another commonly used method (i.e., aloe vera cultivation) in terms of life cycle. The life cycle evaluation showed that the proposed system is economical for several rural areas and may provide opportunities for agriculture electrification and motivate economic growth. 

Roblin [116] presented an irrigation system relying on a solar cell for rural areas where sunlight is often available. The irrigation system includes pumps working on a solar cell instead of an electricity grid and/or a diesel generator. The study showed that the irrigation system based on a solar cell is better than systems that depend on other energy resources because the electricity grid is unavailable at all times, and the operating cost of diesel pumps is high and relies mainly on the diesel price. Although the solar-powered system was relatively expensive, it became a free source of energy after installation in the rural area and proved to be a worthwhile investment in the long term. A similar irrigation system was proposed in [117]. The system adopts photovoltaic panels to power a water pump that converts the continuous drift of water into a drop drift to achieve increased water usage efficiency. 

Kwon et al. [118] suggested a novel prediction technique for energy harvesting with a solar cell within three hours. Moreover, the packet transmissions of the sensor nodes can be controlled depending on the estimated energy. Packet transmissions and energy harvesting are integrated to improve the WSN performance. The integration of these schemes enhances the prediction accuracy and improves the WSN throughput and power consumption. The throughput of the sensor node can be increased by shortening the packet transmission period. By contrast, when the packet transmission period increases, the power consumption of the sensor node decreases. The experimental results show that the proposed packet transmission scheme is superior to the others in terms of data throughput and deadline miss rate. The proposed energy-harvesting method is also predicted more precisely than others by 6.92%.

A study [119] used a solar cell to provide energy to a WSN deployed in a greenhouse. The humidity and temperature of the greenhouse were controlled through a management and monitoring system, which was based on a low-power MSP430 (Texas Instruments, Dallas, TX, USA) microcontroller and an ultra-low-power nRF24L01 transceiver (Nordic Semiconductor, Oslo, Norway). The power consumption of the WSN was minimized as much as possible. In another study, a WSN-based ZigBee (CC2420 module) wireless transceiver and microcontroller (low-power ATmega128L) supplied by the solar cell as a battery charger were considered to monitor the humidity, temperature, rain level, and leaf wetness of vineyards [120]. The WSN covered 30 m between ZigBee units with a low power consumption (35 and 38 mW in transmission and reception modes, respectively). 

#### 3.2.2. Wireless Power Transfer 

Recent developments in wireless power transfer (WPT) are expected to increase the lifetime of WSNs significantly and make them continuously operational, given that WPT techniques can be utilized to transmit electromagnetic energy between transmitter and receiver devices without any contact between the two. WPT is expected to overcome the constraint of the supply of WSNs. Consequently, some researchers highlighted the utilization of mobile node that can deliver power to deployed sensor nodes such as [121,122]. WPT technology poses a challenge to the energy cooperation between neighboring nodes in WSNs. Therefore, in the future, sensor nodes could harvest energy from the environment and transfer this energy to other nodes in the network; in this manner, a self-sustaining network can be achieved [123]. Accordingly, recent research has investigated multi-hop energy transfer [124,125], which paved the way for new energy cooperative schemes and WPT charging protocols.

WPT can be classified into three main subcategories: (i) electromagnetic (EM) radiation, (ii) magnetic resonator coupling, and (iii) inductive coupling, as shown in Figure 2. Wireless charging in WSNs can be performed via EM radiation and magnetic resonant coupling. EM signals suffer from attenuation over distance, and active radiation may pose a threat to the human body. Meanwhile, magnetic resonant coupling can address the power requirements of agriculture based on WSNs because of its efficiency within a distance of several meters. Many studies used WPT to charge sensor nodes for different applications and fields. WPT-based inductive coupling [121] has been used to charge the battery of sensor nodes scattered in agriculture fields. The energy is transferred from the source coil (mounted on a UAV) to the receiver coil (attached with sensor nodes). A 2.4 W transfer power is transferred to charge a single sensor node. The results addressed a number of limitations and challenges related to the charging of sensor nodes via a UAV. In addition, the results revealed that the battery lifetime of the deployed sensors can be significantly extended through the UAV. A similar approach was adopted by Chen et al. [122] to charge a WSN in an agricultural field through the harvesting of electromagnetic radiation.

#### 3.2.3. Air Flow Energy 

Harnessing *wind energy* is another energy-harvesting technique that can be utilized to provide power to sensor nodes in agricultural applications. An adaptive routing protocol, wind energy harvesting, and sleep scheduling were considered in [126] to minimize the power consumption of the ZigBee transceiver and extend the lifetime of the WSN. 

#### 3.2.4. Vibration Energy 

*Vibration energy* based on piezoelectric can be used to charge the battery of sensor nodes to prolong their lifetime. Müller et al. [127] have been analyzed various WSN protocols based on ZigBee (CC2420 and CC2500) and CC1100 with respect to their applicability in the agricultural setting. Furthermore, a fully synchronous protocol with a time-slot architecture for a WSN has been proposed for agricultural machinery to achieve real-time capability, low latency, and deterministic behavior. The objective of their application was to monitor the back door position and filling level of a forage wagon using a ZigBee (CC2420) RF transceiver. Clock synchronization among all nodes is implemented to ensure strict power on/sleep times of each sensor node. Meanwhile, an energy-harvesting unit based on a piezoelectric material has been designed; the unit provides an average power of 200 μW to sensor nodes. In this case, the sensor node can transmit a data payload of nine bytes in 40 ms. 

#### 3.2.5. Water Flow Energy

Multi-energy sources (water, wind, and solar) platform is designed for precision agricultural applications by Morais et al. [128]. The authors explored water flow, wind speed and solar radiation feasible energy sources to encounter the requirements of ZigBee router node in WSN. Thus, several powered solutions for WSN is presented. Design considerations regarding water flow energy, the water flow in the pipes of crops irrigation system are employed to generate the energy for ZigBee router node. This idea can be applied in different agricultural applications such as greenhouses, aquaculture, and hydroponic systems, where the water recirculation in pipes is continuous. Like to bulky hydroelectric generation utilities, a water flow in the pipes, derived from the main water source, can be employed to run a turbine joined to a small direct current (DC) generator. The experimental results revealed that the three energy sources combined together can generate an energy of 58 mAh, more than the requirement of the ZigBee router node (39 mAh). 

#### 3.2.6. Microbial Fuel Cell Energy

Another type of energy harvesting technique is called *microbial fuel cell*. This technique is extracted from energy neutral system. Sartori and Brunelli [129] proposed microbial fuel cell to supply the underground freshwater system that used for monitoring the water level in the phreatic zone, artesian wells, and tanks. The proposed system comprises a low-cost phreatimeter sensor, a low-power microcontroller (i.e., MSP430FR5739), and a low-power LoRa wireless protocol. However, the amount of microbial fuel cell energy extraction of 296 μW is not enough to supply LoRa wireless protocol and microcontroller directly when they are in active mode. Therefore, a DC-to-DC boost is employed to raise the small input voltages of 130 mV to 4.5 V. 

**Figure 3:**
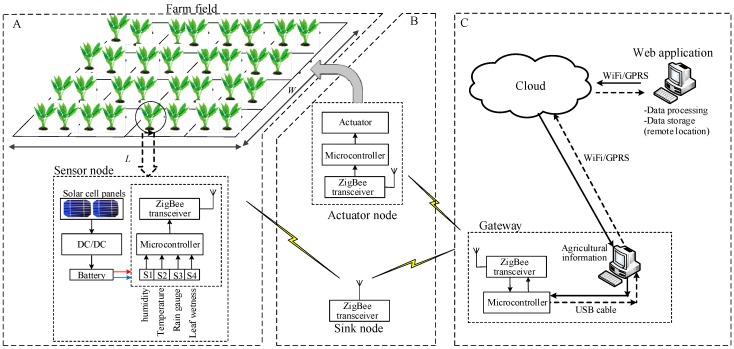
Example of farm field-based Internet of Things (IoT) and provided by a solar cell battery charger: (**a**) Agriculture sensor node with related sensor and solar cell, (**b**) Sink and actuator nodes, and (**c**) Gateway node and cloud computing.

Evidently, most agricultural applications prefer to use solar cells as battery chargers of WSNs because these cells are easy to install, work efficiently when sunlight is available, and supply more energy compared with other energy-harvesting techniques as shown in Table 7. In addition, the table shows most of the previous scholars adopted ZigBee wireless protocol. Clearly, employing ZigBee supports our study that this wireless protocol is more suitable for agricultural monitoring systems. Solar panels can supply 100 mW/cm^2^, whereas radio frequency, thermal, vibration, wind, microbial fuel cell, magnetic resonant coupling WPT, and water flow can supply 0.001, 0.06, 0.8, 1.0, 0.296, 14, and 19 mWs, respectively [128,129,130,131]. For example, as shown in Figure 3 (Part A), if the power consumption of the WSN components presented in [120] is 35 mW for ZigBee (Chipcon CC2420), 0.27 mW for the temperature sensor, 3 mW for the humidity sensor, 0.27 mW for the rain gauge sensor, 5 mW for the leaf wetness sensor, and 24 mW for the ATmega128L microcontroller [132], a single solar cell (2 × 2 cm^2^) panel can adequate supply this WSN. However, when the power consumption of the WSN increases, the dimension of the solar cell panel also needs to be increased.

## 4. Agriculture Requirements for IoT

The IoT represents the visibility of a group of systems, technologies, platforms, and design principles for joining things, depending on the physical surroundings, through the use of the Internet. PA is an application that can employ the benefits of IoT to increase production efficiency, improve the quality of yields, reduce the negative ecological impact, prevent the prevalence of plant-eating pests or plant diseases, alert farmers about farm fires [141], and increase the profitability of several agricultural production schemes. Agriculture involves farming, planting, and animal rearing, and it has grown under the scope of IoT recently [5]. Tracking of animals, monitoring of farms, and irrigation processes are the main domains of IoT for cultivation [142]. Furthermore, feeding, rearing, medication, and vaccination are essential applications of IoT in the agricultural domain [143]. Consequently, several IoT modular architectures have been proposed and implemented by scholars for PA monitoring (as presented in Table 8). Real-time ecological information can be remotely gathered from the agricultural surroundings based on different sensors, which transmit the information to be processed to determine problems, take required actions using actuators, or store data.

Sensors in WSN can be employed to gather information about environmental and physical features, whereas actuators are used to respond to the feedback to control or to perform an action over the conditions (Figure 3, Part B). The use of sensors in agricultural applications poses a number of requirements, including gathering of soil, weather, and crop information; surveillance of agricultural areas, water and fertilizer requirements of diverse pieces of rough land, several crops on a single piece of acreage, different requirements of crops for unlike soil and weather circumstances, and eliminating of interactive solutions and relying on proactive solutions. All these requirements are applied and processed in parallel. Therefore, different sensors and actuators must be used to handle this information and respond to different conditions. Processed information can be utilized to make a decision instead of raw data of the sensor [132]. This information can be transmuted to the cloud computing via gateway for data processing and data storage, as shown in Figure 3 (Part C).

Reference [144] proposed a modular architecture for a wireless sensor node implemented for PA applications. The sensors employed in this modular were soil moisture, temperature, humidity, and wind direction and speed sensors to monitor the agricultural environments. “Adafruit Pro Trinket” platform was selected for the data processing, and nRF wireless protocol was used as a transceiver to transfer sensor data to the gateway (i.e., Intel Edison to reduce the power consumption). The gateway was in charge of collected information from different sensors and passed the information to the cloud server. The proposed system resulted in improved crop quality and minimized negative ecological effect. In [145], the usage of water in smart irrigation systems was controlled based on developed IoT platforms. A cloud agriculture field-monitoring scheme was designed and implemented to enable the farmer to monitor the soil condition. Soil moisture and weather temperature were monitored to automate the irrigation system in farm while minimizing water consumption, which led to reduced cost and maintained environmental benefits. The proposed water irrigation system included three main parts: a WSN and actuator, a cloud platform, and user application. The WSN and actuator part consisted of sensor nodes for collecting the soil moisture and weather temperature, an actuator node to control the irrigation process, and a sink node that could pass all farm information to the cloud platform. All sensor, sink, and actuator nodes adopted low-power ZigBee wireless protocol.

WSN and 3G network-based agriculture IoT was considered in [146] to monitor the air temperature, air humidity, and soil moisture. The entire system consisted of sensor nodes, actuators sink nodes, server, gateway, and the mobile phone device of the farmer. The collected information by the sensor nodes was sent to the server via gateway and Internet network. The gateway connected the WSN with the Internet to relay the command control among the sensor nodes, the phone of the farmer, the actuators, and the server. However, in this system, the data transmission over the 3G network must ensure real-time communications to support the agricultural WSN.

IoT-based agriculture applications changed classical agricultural surveillance approaches by speedily providing quantitative data with significant temporal and spatial resolution. Several IoT platforms have recently been available in the market. For instance, in agricultural applications, SmartFarmNet [147] platforms can be used to provide more than 30 packages for good commercial products, such as Libelium [45], and experimental, such as Motes [148] and Arduino [149] sensor platforms. Other examples of agricultural applications based on IoT are provided in Table 8. The table presents a comparative analysis of several scholars in terms of sensors, actuators, IoT devices, and IoT platforms that can be used to support agricultural applications. 

The major requirements of PA domain such as sensors, microcontrollers, and wireless technologies are highlighted and classified in Table 9. For better clarification, the sensors are classified into three main categories leaf wetness/plant, soil moisture, and environment sensors [5,132]. These sensors can be utilized in several potential agriculture applications. In Table 9, the microcontroller/processor and wireless protocols are also highlighted. Among the microcontrollers, the majority of agriculture sensor nodes adopting ATmega128L because this microcontroller supports data aggregation, decision making, storage capability, flash memory, low complexity, and energy management through multi-levels of power down modes [132]. ZigBee and LoRa wireless protocols/technologies are widely used in several agricultural applications, as discussed previously in Section 2. 

In precision agriculture, to obtain high-quality products, the environmental parameters should be controlled to provide optimal values. For example, the temperature rate that has a positive effect on the growth of the pepper field is between 20 and 25 °C. The temperature in rainy, sunny, and nightly days should be kept between 20 and 22 °C, between 25 and 28 °C, and between 18 and 20 °C, respectively and the minimum value should be not lower than 16–17 °C. Air humidity for pepper vegetables should be kept between 60% and 70%. The high-temperature value combines with low humidity value may be caused a negative influence on the growth of the pepper. The maximum humidity value in pepper greenhouse should be not above 70%. Soil pH (i.e., soil acidity) is another factor that directly effects on the pepper crops, where it can measure the quantity of calcium in the soil. The soil for pepper crops can be acid or alkaline depending on the weather conditions. It should be acid for wet climate and alkaline for a dry climate. Soil with pH levels above 7.0 is considered alkaline, while soil with pH levels below 7.0 has acid nature. Soil pH values must be controlled according to the type of the agricultural yield. For pepper crop, the best values for pH gauge are between 6.5 and 5.5. Therefore, the humidity, temperature, and soil pH should be constantly supervised in order to identify the right time for draining and watering the agricultural crops. 

## 5. Challenges and Limitations

The development of WSNs prompted new research trends in the agricultural domain. Micro electromechanical system (MEMS) technologies allow for the manufacture of cheap and small sensors. The pervasive nature of the process, together with tiny, self-regulating sensor nodes, cost-effective equipment, and scalability, indicate that WSNs can be used for agriculture automation [5]. However, regarding the distribution of WSNs to observe different agricultural climates, a number of open challenges and limitations remain. Several of these challenges and limitations in existing agricultural applications based on WSNs are listed below together with suggestions on how to address them.

(1) *Power consumption and battery life*: A WSN consists of three main components: sensors, microcontrollers, and RF transceivers. Given that the battery of a sensor node provides limited energy, ensuring that the components of the sensor node consume minimum power is crucial. In particular, reducing the power consumption of the RF transceiver, which consumes more power than the other components in a sensor node, would alleviate this problem [67,127]. Moreover, this issue can be addressed in two steps. The first is to propose an intelligent energy-efficient algorithm. The second step can be performed by utilizing available energy-harvesting techniques, such as solar cells, vibration, and WPT.
(2) *Communication range*: WSNs suffer from the effect of harsh ecological conditions because of the wide range of open agricultural surroundings [5]. The WSN protocol contains mechanisms to resist the effect of data transmission failures in the network, which increase due to ecological effects. In agricultural applications, most wireless sensor technologies support a relatively short communication range. Therefore, many sensor and router nodes need to be diffused in a WSN. In the point-to-point Zigbee network, the communication distance can reach 100 m in outdoor environments. The ZigBee communication range can be extended by adopting multi-tire, ad-hoc, decentralized, and mesh network topologies. Drones or UAV also can be used as a mobile router node to extend the communication range within a farm field. A drone could pass the collected data from the sensor nodes to the master node through multi-hop. However, using drones entails other challenges and limitations.
(3) *Propagation losses*: In agricultural applications, WSNs must be able to work in diverse surroundings, such as ground, bare land, orchards, greenhouses, farms, and complex topography; they must also be able to operate in all climate conditions. All these conditions influence the performance of radio propagation. Whether the topography is simple or complicated, the communication possibility between the points in a WSN still suffers from serious challenges. The signal transmitted from the sensor nodes in agricultural applications need to pass through a heavy crop canopy to arrive to at the receiver nodes, which cannot ensure a sufficient clearance area and will cause signal propagation absorption, reflection, attenuation, and scattering. In this case, the link quality is degraded, especially when the signal spreads through dense crops. Therefore, when deploying WSNs, communication link quality and temporal and spatial variables must be guaranteed. The communication performance of WSNs is related to the working surroundings. Therefore, due to the limited resources and power budget of WSNs, an accurate wireless channel path loss model must be adopted to reflect the propagation features. This model is expected to demonstrate correct optimization and network evaluation performance throughout the deployment design process to develop the energy efficiency of the nodes [15], improve the target detection and localization applications [16], decrease the number of retransmission, and ensure Quality of Service (QoS) of the network [175].
(4) *Routing*: Different problems can emerge due to packet collision and limited bandwidth, which are introduced by channel propagation, and so on. Therefore, when a WSN is deployed in a wide area in farm fields, multi-hop is required. Kim et al. [3] developed an independent mobile robot platform based on a mobility task for surveillance to overcome channel interference.
(5) *Localization and tracking*: Tracking and localization of a herd of cattle are considered smart farm applications based on WSNs. For example, a WSN can be employed to track and localize of dairy cows to enable herd management. ZigBee wireless protocol is used to monitor animal locations and behaviors, such as walking and standing, lying down, and grazing [16,18]. In this context, several considerations, such as radio interference, animal situation, and mobility, changes in WSN topology, penetration depth of the signal through the animal body, height of the collar, and access point antennas, need to be taken into account [176]. These considerations pose challenges in the localization and tracking of the animals.
(6) *Reliability*: Agricultural monitoring systems based on different environmental sensors can also be used to monitor pollution aside from climate conditions. Important information on climate conditions is reported to related agencies and farmers from a remote location for advance investigations. Dangerous information needs to be dealt with immediately in an emergency, which means that data transmission in WSNs should demonstrate high reliability [3].
(7) *Scalability*: In agricultural applications, the construction of WSN-based fault-tolerant and robust hierarchical architectures requires large-scale deployment relative to single-level network architectures. A hierarchical architecture can be scaled up for developing applications by duplicating to several fields. In this context, to increase the number of WSNs over a vast area, multiple wireless router nodes are placed in an agricultural field to guarantee sustained operation.
(8) *Cost*: The total hardware and software costs of sensor nodes are important. The design of sensor nodes for any agricultural application level must involve a low cost while demonstrating a robust performance; moreover, the design needs to be available for use by poor country markets [5]. This challenge can be overcome by reducing software and hardware costs further.
(9) *Real time*: Most crops are vulnerable to climate conditions, such as humidity, intensity of illumination, and temperature. This vulnerability is a burden to farmers who monitor changes in climate conditions hourly and/or daily because doing so is labor-intensive. Moreover, in greenhouses, a fire can occur and lead to severe agricultural disasters. This evidence suggests that the monitoring of the ecological conditions of agricultural WSNs needs to be in real time. Real-time monitoring will enhance yield production and plant growth and avoid dangerous disasters in farms [3].
(10) *Storage and recording of data*: Large amounts of data are recorded from agriculture observing systems because several WSNs in agricultural applications contain several sensors for crop growth analysis and harvesting estimation. This condition requires the base station to monitor changes in farm fields by analyzing patterns. The base station must thus be supported by a high storage capacity.
(11) *Security*: Security and protection are important issues in agricultural products. Protection from insects or attacks of rodents in grain stores or fields is essential. Such a challenge must be considered to maintain the security level of agriculture. Protection and security can be achieved based on real-time analysis and processing of agricultural information without human intrusion [159].
(12) *Delay tolerance*: Critical delay poses a challenge to agricultural applications. Some agricultural applications can be considered as time sensitive, such as those used in farm fire detection, detection of exposure of crops to pests, cow heat event detection during milking, and exposure of cattle to heat for a certain period. Such information must be transmitted as fast as possible if the critical issue is to be handled. In such a case, a tradeoff between energy consumption and data timelines is necessary. By contrast, some agricultural applications are delay tolerant (i.e., time insensitive), such as protein content, milk fat [177], gathering data from soil and grass monitoring. With delay tolerant approach, the short range low power wireless protocols can be used [178]. Where the information of the agricultural field can be collected and transferred to the master node or to a cloud computing node using “messengers” e.g., a mobile node (mobile robot or UAV). Consequently, the cost [179] and the complexity of the agriculture WSN will be reduced and the lifetime will be increased.
(13) *Fault tolerance*: Fault tolerance is a crucial feature of WSNs for succeeding PA. Several faults may occur in PA based on the WSN system, which are (i) communication failure, (ii) faulty sensor setting, (iii) sensor component faults producing incorrect value, and (iv) node failure because of exhausted battery or any other cause. Gutiérrez et al. [40] introduced communication failure and node fault tolerance for an irrigation system. If any fault occurs, the irrigation system tracks the default irrigation program. The node energy depletion failure was minimized in [1,40,180] by adopting solar-cell-powered nodes. Data aggregation and topology control schemes are probable to be fault tolerant for deploying sensor nodes in a vast area.
(14) *Data management*: The data management in agriculture poses a challenge because the large amount of data that can be collected from several sensors spread in agricultural field, especially when the agricultural data are intended to be connected to the cloud. Determination of the (i) data analysis method, (ii) data collection schemes, (iii) sensor types, (iv) semantic sensor networking, (v) big data, and (vi) complex event processing enables the designer to manage these crucial aspects. The integration of IoT and software-defined network also introduces a promising and new methodology in deployment, monitoring, and design of network services and resources [181].
(15) *Heterogeneous sensors:* Integration of wired and wireless heterogeneous sensors into information platforms to perform interoperability pose challenges in PA. Chen et al. [152] proposed “web service-enabled cyber-physical infrastructure” to solve this issue. The proposed system was able to integrate, process, acquire, and distribute surveillance data from different physical sensors spread in the PA system over the Internet. The infrastructure was executed to serve as an architecture middleware between PA clients and heterogeneous sensors.

## 6. Conclusions

A review of WSN-based agricultural applications was presented. A comparison was conducted among different wireless technologies or protocols, such as WiFi, Bluetooth, ZigBee, GPRS/3G/4G, LoRa, and SigFox. The comparison results revealed that the ZigBee and LoRa wireless protocols are more convenient for agricultural applications than the others because of its low power consumption and suitable communication range for ZigBee and long for LoRa. A classification of energy-efficient techniques or algorithms and energy-harvesting techniques was also provided. Based on the presented taxonomy, we show that significant types of energy-efficient and energy-harvesting techniques can be used in the agriculture domain. Previous research was also investigated and compared to explore the current problems in agricultural applications based on WSNs and obtain optimum solutions for maintaining system performance. Challenges and limitations were introduced for design considerations in the future. The state-of-the-art approaches of IoT in agricultural applications were reviewed and compared to explore various sensors, actuators, devices, IoT platforms, and application layers.

## Figures and Tables

**Table 1:** Different wireless communication technologies.

Parameters	ZigBee	Classic BT	BLE	WiFi	GPRS	LoRa	SigFox
Standard	IEEE 802.15.4	IEEE 802.15.1	IEEE 802.15.1	IEEE 802.11a,b,g,n	N/A	IEEE 802.15.4g	IEEE 802.15.4g
Frequency band	868/915 MHz and 2.4 GHz	2.4 GHz	2.4 GHz	2.4 GHz	900–1800 MHz	869/915 MHz	868/915 MHz
Modulation type	BPSK/OQPSK	GFSK, DPSK, and DQPSK	GMSK	BPSK/OQPSK	GMSK/8PSK	GFSK	DBPSK(UL), GFSK(DL)
Spreading	DSSS	FHSS	FHSS	MC-DSSS, CCK	TDMA, DSSS	CSS	N/A
Number of RF channels	1, 10, and 16	79	40	11	124	10 in EU, 8 in US	360
Channel bandwidth	2 MHz	1 MHz	1 MHz	22 MHz	200 kHz	<500 KHz	<100 Hz
Power consumption in Tx mode [5,40,56,59,60,61]	Low	Medium	Ultra-low	High	Medium	Low	Low
	36.9 mW	215 mW	10 mW	835 mW	560 mW	100 mW	122 mW
Data rate	20, 40, and 250 kbps	1–3 Mbps	1 Mbps	11–54 and 150 Mbps	Up to 170 kbps	50 kbps	100 bps
Latency [62,63,64,65]	(20–30) ms	100 ms	6 ms	50 ms	<1 s	N/A	N/A
Communication range [48,66]	100 m	10–50 m	10 m	100 m	1–10 km	5 km	10 km
Network size	65,000	8	Limited by the application	32	1000	10,000 (nodes per BS)	1,000,000 (nodes per BS)
Cost [5]	Low	Low	Low	High	Medium	Low Cost	Low Cost
Security capability	128 bits AES	64 or 128 bits AES	64 or 128 bits AES	128 bits AES	GEA, MS-SGSN, MS-host	AES 128b	Encryption not supported
Network Topologies	P2P, tree, star, mesh	Scatternet	Star-bus	Point-to-hub	Cellular system	Star-of-stars	Star
Application	WPANs, WSNs, and Agriculture	WPANs	WPANs	WLANs	AMI, demand response, HAN	Agriculture, Smart grid, environment control, and lighting control	Agriculture and environment, automotive, buildings, and consumer electronics
Limitations	line-of-sight (LOS) between the sensor node and the coordinator node must be available	Short communication range	Short communication range	High power consumption and long access time (13.74 s)	Power consumption problem	Network size (scalability), data rate, and message capacity	Low data rates

**Table 2:** Sleep/wake schemes for previous research in agricultural applications.

Power Reduction Scheme		Reference Example	Wireless Protocol/Device	Power Savings/Battery Lifetime	Communication Distance/Field Size	Sensors/Actuators	Application	Limitations
**Sleep/wake scheme**	Duty-cycle	[8]/2016	LoRa	4408 h	Limited	Soil temperature, Soil moisture, air temperature, air humidity and light intensity/alert messages	Greenhouse	Communication distance
		[38]/2013	WiFi	9.5 days	100 m	Temperature, humidity, water level, soil moisture, light, and pressure	Precision Agriculture	Short battery life
		[42]/2015	ZigBee and GSM/GPRS	13.35 days	Long	Soil moisture, temperature, pressure, and water electrical conductivity and temperature	Irrigation	Short battery life
		[49]/2013	ZigBee and GSM/GPRS	21 days	20 m	Air temperature, air humidity, and solar radiation	Vineyard	Short battery life and communication distance
		[69]/2013	GPRS	Low power	30 m	Soil moisture/sprinkling machine	Precision Agriculture	Data losses-Measurement error
		[70]/2013	ZigBee and GPRS	Low power	23 m	Temperature and soil moisture/solenoid valves	Precision Agriculture	Conflicted in communication between ZigBee and GPRS
		[71]/2014	DZ50 (RFM12b)	700%/7 years	Short	Soil moisture/solenoid valves	Precision Irrigation	Low data rate
		[72]/2015	ZigBee and GPRS/3G	8.1 days	2000–3000 m	Wind speed, wind direction, temperature, humidity, rain gauge, water and pH level	Crop fields	Short battery life
		[78]/2012	ZigBee (CC2530)	150 day (3606 h)	400 m	Soil moisture, ambient temperature, soil temperature, and ambient humidity/irrigation equipment	Agriculture/farm field	RSSI measurements are not considered the actual field
		[79]/2014	ZigBee (CC2530)	84.9 h	65, 95, 200 m	Soil moisture, air humidity, and air temperature/irrigation system	Orchard, greenhouse, and farmland	Packet losses
	MAC protocol	[39]/2011	Simulation	10%	1000 m	Temperature, and soil moisture/solenoid valve and motor	Irrigation	High power consumption in the case of the sensor nodes far from base station
		[73]/2013	IEEE 802.15.4 (CC2420)	745.4 days	50 m	Temperature, light intensity, and humidity	Greenhouse agriculture	Short communication distance
		[74]/2013	ZigBee	6.5 month	10 m	Air temperature, soil pH, humidity light intensity, and soil moisture/irrigation system	Precision farming	Proposed protocol have additional complexity
		[75]/2011	IEEE 802.15.4 (CC2420)	222 and 1204 days	84 m	Air temperature and soil moisture/drip water system	Precision horticulture	Gateway consumes more power because it is always awake
		[76]/2010	IEEE 802.15.4 (CC2420)	Low power	50 m	Leaf temperature and wetness and air temperature and humidity/relay	Greenhouse	The power consumption of the sensor node increases with the number of sensors
	Topology control	[77]/2016	ZigBee	Low power	100 × 100 m^2^	Soil moisture, temperature and humidity/valve	Irrigation	More power is consumed at long communication distance

**Table 3:** Radio optimization schemes for previous research in agricultural applications.

Power Reduction Scheme		Reference Example	Wireless Protocol/Device	Power Savings/Battery Lifetime	Communication Distance/Field Size	Sensors/Actuators	Application	Limitations
**Radio optimization scheme**	TPC	[82]/2010	CC1110 module	≈10%	50 × 50 m^2^	Agricultural environment sensors	Precision agriculture	Large power is consumed through the wakeup synchronization
		[83]/2012	ZigBee CC2420	8.5%	180, 66, and 60 m	Agricultural environments sensors	Agriculture	Simulation study and did not implement in the real environments
	Cognitive radio	[19]/2012	ZigBee-Pro	14 years	Long	Temperature, light intensity, humidity/humidifier, heater, and ventilation	Greenhouse	Limited to one topology (i.e., star topology) to save power
	Modulation scheme	[88]/2016	Simulation	52% (MFSK), 55% (MSK)	10, 30, and 100 m	Different applications including agricultural sensor network	Suggested to use in agriculture application	Power consumption increases with communication range

**Table 4:** Data mitigation schemes for previous research in agricultural applications.

Power Reduction Scheme		Reference Example	Wireless Protocol/Device	Power Savings/Battery Lifetime	Communication Distance/Field Size	Sensors/Actuators	Application	Limitations
**Data mitigation Scheme**	Data gathering	[17]/2016	ZigBee and GSM/GPRS	22%	Short	Temperature, illumination, CO_2_ rate, and humidity/heating, ventilation, dehumidification, and humidification	Greenhouse	System complexity due to fuzzy logic algorithm is implemented in FPGA
		[92]/2014	IEEE 802.15.4 (CC2420)	58.8%	50 and 100 m	Temperature and moisture	Precision agriculture	Limited computational capacity of the sensor nodes
		[93]/2011	ZigBee and GPRS	Low power	Less than 52 m	Soil moisture and temperature, soil electrical conductivity, and environmental temperature and humidity/irrigation system	Smart farming	Path losses due to obstacles
	Data compression	[41]/2010	CC1000 RF module	359 days	150 m	Soil moisture/drip water system	Auto-irrigation	When new node added to the network, encoding all nodes again is necessary
	Data-driven	[95]/2017	WiFi (ESP8266)	81.53% (SPIN) and 36.84% (ESPIN)	45 m (150 feet)	Air temperature, soil moisture, air humidity, and light intensity	Precision agriculture	The sensor node stays awake until received message from relay node, this process wastes power consumption
	Data rate	[98]/2012	IEEE 802.15.4 (CC2420)	150%	N/A	Soil water potential, soil moisture and temperature	Sap flow, soil moisture, and soil water	Under low battery power (i.e., 2.3 V) of the sensor node, the proposed algorithm becomes invalid

**Table 5:** Routing protocol schemes for previous research in agricultural applications.

Power Reduction Scheme		Reference Example	Wireless Protocol/Device	Power Savings/Battery Lifetime	Communication Distance/Field Size	Sensors/Actuators	Application	Limitations
**Energy-Efficient routing scheme**	Sink mobility	[99]/2016	Simulation	High power	672 m	Environmental Sensors for monitoring forest zones	Forest area	Packet losses leads to more energy consumption
		[100]/2016	IEEE 802.15.4	N/A	40 m	Agricultural environments sensors	Precision agriculture	Using predefined paths have many disadvantages: -break of the WSN operation -the existence of an obstacle leads to obstruct WSN operation
	Multi-path routing	[67]/2015	ZigBee	1825 min	150 m	Soil humidity, soil temperature, and air speed/mechanical and hydraulic system	Irrigation system	TEEN protocol consumes a lot of power at long communication distance
		[68]/2016	ZigBee (CC2530)	30%	Less than 200 m	Shadow detection, temperature, and humidity/shadow tracking to save energy	Trees in the agriculture field	Solar cell system is generally irregular and extensively influenced by the change of weather
		[101]/2012	Crossbow Technology (based on IEEE 802.15.4) and 3G	4 weeks	1000 m	Soil moisture, rain gauge, water content, soil suction, and soil temperature/water pump	Irrigation system	N/A
	Cluster architecture	[102]/2014	ZigBee	20 times traditional without cluster heads	180 m	Air temperature, soil water potential , soil moisture, and humidity	Crop farming	Unreliable communication beyond 80 m
		[103]/2013	Simulation	N/A	150 m	Area of interested sensors	Agriculture	ECHERP routing protocol did not take into consideration the time constraints and Quality of Service (QoS)
		[105]/2012	ZigBee (CC2530)	Low power	50 m	Temperature, light, CO_2_ concentration, and humidity	Greenhouse	Time synchronization accuracy
	Routing metric	[104]/2015	IEEE 802.15.4 (CC2520)	28.4 days	100 m^2^	Light intensity and air temperature	Precision agriculture	Short battery life

**Table 6:** The combination of power reduction schemes for previous research in agricultural applications.

Power Reduction Scheme		Reference Example	Wireless Protocol/Device	Power Savings/Battery Lifetime	Communication Distance/Field Size	Sensors/Actuators	Application	Limitations
**Combination schemes**	TPC and MAC and routing protocols schemes	[106]/2011	RF transceiver (CC1100)	65%	N/A	Crop growth, carbon cycle, and hydrologic flow/irrigation system	Precision agriculture	Fault management detection and improving are not considered in the work
	Encoding and modulation schemes	[107]/2011	IEEE 802.15.4 (CC2420) and RF transceiver (CC1100)	53%	N/A	Data generated by the sensors as ASCII text	Agriculture	Low data rate
	Cluster architecture with TDMA-based MAC protocol and data aggregation schemes	[108]/2016	IEEE 802.15.4 (CC2420)	3–5 times traditional	100 m	Farm environment sensors	Agricultural environments	The number of clusters based on LEACH does not converge 100 × 100 m^2^, which reduces the lifetime of WSN network
	Data acquisition, compression, and sampling schemes	[109]/2014	C1110 RF module	8 days	N/A	Leaf wetness, humidity, camera, and temperature	Vineyard	Communication range is limited due to omnidirectional antenna of the RF module

**Table 7:** Energy-harvesting techniques for previous research works used in agricultural applications.

Energy Harvesting Techniques		Reference Example	Wireless Protocol/Device	Harvesting Energy/Power/Power Saving	Sensors/Actuators	Applications	Limitations
**Solar Energy**	Solar cell	[40]/2014	ZigBee (XBee-Pro S2) and GPRS	240 mW	Temperature, soil moisture/solenoid valve	Irrigation system	The solar cell can only charge three batteries type AA 2000 mAh Ni-MH
		[52]/2015	RFD 900 (902–928 MHz)	1.75–3 W	CH_4_ and CO_2_ Concentration/solenoid valve and motor	Greenhouse gases	Power consumption of drone and solar cell weight and size may restrict flight endurance
		[68]/2016	ZigBee (CC2530)	500 mW	Shadow detection, temperature, and humidity/shadow tracking to save energy	Trees in the agriculture field	Solar cell system is generally irregular and extensively influenced by the weather changes
		[72]/2015	IEEE 802.15.4 and GPRS/3G	2 W	Wind speed, wind direction, temperature, humidity, rain gauge, water and pH level	Crop fields	The battery supports the sensor node for seven days only
		[109]/2014	C1110 RF module	500 mW	Leaf wetness, humidity, camera, and temperature	Vineyard	Communication range due to omnidirectional antenna of the RF module
		[119]/2010	nRF24L01	N/A	Temperature, pressure, humidity, vibration, and flow/irrigation system	Greenhouse	The communication range (100 m) becomes unstable when there are other communications in the same area or when the people moving in the communication path
		[120]/2012	IEEE 802.15.4 (CC2420)	1 W	Temperature, leaf wetness, rain gauge, and humidity/condensation or infiltration system	Vineyard	Solar energy changes with time
		[133]/2015	RFD 900 (902–928 MHz)	59.14 Wh	CH_4_ and CO_2_ Concentration/gas chamber and olenoid valve	Greenhouse	The maximum area for the solar cell panels is restricted by the UAV wings size
		[134]/2012	ZigBee (Mica2 motes)/GPRS	20 W	Air humidity, air temperature, soil moisture, and soil temperature/irrigation system	Agricultural environments	Single antenna is not suitable for both point-to-point links and broadcast
		[135]/2015	WiFi (IEEE 802.11a)	180 mW (sunny area), 24 mw (shady area)	Temperature and humidity/shadow tracking to save energy	Agricultural environments	The intensity of solar energy changes with weather conditions and shadow (depending on height of crops, time, and orbit of the sun)
		[136]/2010	IEEE 802.15.4	2 W	Temperature, light, humidity, and wind speed	Agricultural and forest ecology	Due to dense forests, the solar cell can not supply the sensor nodes more than two hours
**WPT**	Inductive coupling	[121]/2016	Zigbee	2.4 W	Vibration, pressure soil moisture, and temperature	Agriculture fields	Strong coupled magnetic resonance are required
	Magnetic resonant coupling	[137]/2015	N/A	1315 J	Agricultural environments sensors/water processing system	Agriculture areas	Exhausting the UAV battery
	Electromagnetic wave	[122]/2016	Zigbee	N/A	Temperature, Strain, humidity, and displacement	Agriculture fields	Harvested energy is inadequate to replenish an ad hoc network with multi-hop
**Air Flow Energy**	Wind turbine	[126]/2014	Zigbee	70–100 mW	Ambient temperature, rain fall, and soil moisture/irrigation system	Vineyard	Wind power is inefficient when the wind intensity is not constant and irregular
**Vibration Energy**	Piezoelectric convertors	[127]/2010	ZigBee (CC2420 and CC2500) and CC1100	200 µW	Ambient vibration sensor	Agricultural machinery	Transmission errors due to Interferences from similar neighboring WSN and third-party system
		[138]/2016	IEEE 802.15.4 (CC2500)	14%	MEMS inertial	Agricultural machinery	N/A
		[139]/2011	IEEE 802.15.4	724 μW@2.0g	Vibration sensor	Agricultural machinery	Duty-cycle of the end device must be modified according to the total power collected by the piezoelectric convertor
**Thermal Energy**	Thermoelectrical elements	[140]/2012	ZigBee (CC2530 embeded in HaLOEWEn platform	N/A	Temperature and soil moisture/irrigation control system	Precision irrigation	Harvested energy is comparatively low based on thermoelectric element
**Water Flow Energy**		[128]/2008	ZigBee	16–19 mW	Soil moisture, air temperature, relative humidity, soil temperature, and solar radiation/irrigation control system	Precision Agriculture	The amount of energy harvested is not enough alone to supply the ZigBee router node
**Microbial Fuel Cell Energy**		[129]/2016	LoRa	296 μW	Capacitive phreatimeter/irrigation system	Precision agriculture	The amount of microbial fuel cell power is not enough to power the LoRa wireless protocol and microcontroller directly

**Table 8:** Sensors, actuators, and platforms used in agricultural applications based on IoT.

Reference Example	Sensors/Actuators	IoT End Device Wireless Protocol	IoT Platform/Device	IoT Application Layer
[8]/2016	Soil temperature, Soil moisture, air temperature, air humidity and light intensity/alert messages	LoRa	LoRaWAN	User interface, remote monitoring, and email
[42]/2015	Soil moisture, temperature, pressure, and water electrical conductivity and temperature	ZigBee	GSM/GPRS	Web application (HTML5, PHP, and Javascript)
[95]/2017	Air temperature, soil moisture, air humidity, and light intensity	WiFi (ESP8266)	WiFi	Web services
[143]/2016	Camera to monitor the rice leaf disease	Sensor networks	Wisekar and cloud Computing	Web application and user-defined
[144]/2017	Temperature, humidity, soil moisture, and wind direction and speed	nRF wireless protocol	Intel Edison and cloud computing	User interface and ustom server
[145]/2015	Temperature and soil moisture/electrovalve	eZ430-RF2500 (IEEE802.15.4/ZigBee-based CC2500)	WiFi 802.11 or GPRS through http and Cloud computing	Web applications
[147]/2016	Temperature and soil moisture/irrigation system	Libelium WaspMotes, Remote, Netatmo, etc.	SmartFarmNet and Cloud computing	Server application, user interface, and do-it-yourself visualization
[150]/2015	Temperature, humidity, light, pressure, camera, CO_2_, and wind direction and speed/air flow, sprinkler, and sunlight secreen	ZigBee	Ethernet shield and GPRS	User applications and server applications
[151]/2015	Ambient temperature, soil moisture, pH value, and humidity/valves and pumps	ZigBee (XBee)	Ethernet/WiFi/GSM	User applications
[152]/2015	Air temperature, wind speed/direction, air humidity, air pressure, net radiation, sunshine duration, and precipitation/irrigation system	IEEE 802.11 or Bluetooth	GPRS	User applications (desktop client, web client, and mobile client) and web processing service
[153]/2015	Pesticide concentration sensor	Hypogynous computer	Epigynous computer	HTML files, Webpage, and Smartphone
[154]/2016	Air temperature, relative humidity, solar radiation, precipitation, water, and nutrients/irrigation system	IEEE 802.15.4/ZigBee	FIWARE platform and cloud computing	Web services, data analysis, and database
[155]/2016	Temperature, Luminosity, PH, moisture, EC/lamps, electro-valves, and pumps	IEEE 802.15.4/ZigBee	Cloud computing	Web services, data analysis, database, and HMI interfaces
[156]/2016	Temperature, light intensity CO_2_ concentration, and humidity	ZigBee (CC2530)	GPRS (SIM300 module)	User application
[157]/2016	Soil moisture/water pumps, fan, and mist	ZigBee (XBee)	WiFi and GSM/GPRS	Graphical user interface
[158]/2016	Air temperature, wind speed and direction, leaf wetness, soil moisture, air humidity, rain volume/fertilizers or spraying chemicals and watering system	nRF24L01	IEEE 802.11b/g/n (WiFi) and Cloud computing	Data visualization, data storage, data analysis, and application program interface
[159]/2016	Web camera, ultrasonic ranging, infrared heat sensor, and ultrasonic sound repeller	Wired connection to PC	PTC’s ThingWorx’s	User application, web services, and http and several Internet protocols
[160]/2016	Temperature, humidity carbon dioxide, soil moisture light intensity, and pH value	Bluetooth and mobile device	4G and cloud computing	Intelligent management (neural network)
[161]/2016	Ultrasonic, air humidity, air temperature, LDR sensor, and soil moisture/pumps, solenoid valve, fogger system, lights, and peltier	RFID tags	GSM sim900a and WiFi	SMS, e-mail, google spreadsheet, e-commerce website
[162]/2016	Air temperature/fan, curtain, and shutter	ZigBee	GPRS	Web applications
[163]/2016	Temperature, soil moisture, light, and humidity/water pump	nRF24L01	GPRS/GSM	Microsoft active server pages and MYSQL
[164]/2016	Relative humidity, barometric pressure, temperature, light intensity, camera, and proximity sensing/buzzer, SMS alerts	IEEE 802.15.4/ZigBee	Wireless connection	Web application, Android application, and cloud storage
[165]/2016	Temperature	Off-the-shelf mesh WSN	SmartMesh IP Manager	Server applications and database
[166]/2016	Air temperature, illumination intensity, and relative humidity	Mica2 (CC1000)	2G/3G and cloud computing	User applications (display terminal, PC, PDA, remote monitoring device) and GUI
[167]/2017	Temperature, illumination, camera, and humidity/vaporization system	ZigBee	WiFi	Data mining, data inquery, and data storage
[168]/2017	Liquid level sensor/water pump	Wired connection	Ethernet shield	User-defined
[169]/2017	Temperature and light	IEEE 802.15.4	IEEE 802.11g, 802.11a, and RFS 6000	User applications and server applications
[170]/2017	Temperature, pH value, and Oxygen	ZigBee	GSM, WiFi, and cloud computing	Web services, desktop application, and mobile applications
[171]/2017	Relative humidity	LoRa/LoRaWAN	GPRS/3G/4G and cloud computing	Liquid crystal display (LCD)
[172]/2017	Relative humidity, temperature, air pressure, and luminosity	WiFi	IBM Watson IoT, IBM Bluemix cloud service, and cloud computing	data storage, and data graphs and visualized
[173]/2017	Soil moisture, salinity, and temperature	WiFi/ZigBee/Bluetooth	WiFi, cloud	HTTP protocol and smartphone applications
[174]/2017	Soil moisture, soil pH, and camera	WiFi and drone	FarmBeats (based Ethernet or WiFi) and cloud computing	Web interface (data access, cross-farm analytics, and long term applications)

**Table 9:** Types of sensors, microcontrollers, and wireless protocols/technologies used in precision agriculture (PA).

Sensors			Microcontrollers	Wireless Protocols/Technologies
Soil Related	Leaf/Plant Related	Environment Related		
Hydra probe II soil sensor (www.stevenswater.com) MP406 (www.ictinternational.com.au) Pogo portable soil sensor (www.stevenswater.com) ECH_2_O EC-5 (http://www.decagon.com) ECRN-50 low-REC (http://www.decagon.com) WET-2 (http://www.dynamax.com) EC-250 (http://www.stevenswater.com) BetaTherm 100K6A1B (http://www.campbellsci.com) Waspmote (http://www.libelium.com/products/waspmote/) VH-400 (http://www.vegetronix.com) THERM200 (http://www.vegetronix.com) Tipping bucket rain gage (http://www.stevenswater.com) AquaTrak 5000 (http://www.stevenswater.com) ECRN-100 high-REC rain Gauge (http://www.decagon.com) 107-L temperature Sensor (BetaTherm 100K6A1B Thermistor) (http://www.campbellsci.com)	237-L, leaf wetness sensor (http://www.campbellsci.com) Leaf wetness sensor (http://www.decagon.com) LW100, leaf wetness sensor (http://www.globalw.com) TT4 multi-sensor thermocouple (www.ictinternational.com.au/thermocouple.htm) SenseH2™ hydrogen sensor (http://www.ntmsensors.com) LT-2 M (leaf temperature) (http://www.solfranc.com) TPS-2 portable photosynthesis (www.ppsystems.com/Literature/EDSTPS2_System.pdf) PTM-48A photosynthesis monitor (http://phyto-sensor.com/PTM-48A) Cl-340 hand-held photosynthesis (http://www.solfranc.com) 107-L (BetaTherm 100K6A1B thermistor) (https://www.campbellsci.com) YSI 6025 chlorophyll sensor (http://www.ysi.comysi_6025.pdf) Field scout CM1000TM (http://www.specmeters.com/pdf/2950FS.pdf)	Met station one (www.stevenswater.com) CM-100 compact weather station (http://www.stevenswater.com) CS300-L Pyranometer (http://www.campbellsci.com) HMP45C (http://www.campbellsci.com) SHT71 (http://www.sensirion.com/humidity) LI-200 Pyranometer (http://www.stevenswater.com) XFAM-115KPASR (http://www.pewatron.com) Cl-340 (http://www.solfranc.com) SHT75 (http://www.sensirion.com/humidity) Met One Series 380 rain gauge (http://www.stevenswater.com) Waspmote (http://www.libelium.com/products/waspmote/) WXT520 compact weather station (http://www.stevenswater.com) All-In-One (AIO) Weather Sensor (http://www.climatronics.com) RM Young (model 5103) (http://www.stevenswater.com) RG13/RG13H (http://www.vaisala.com) SHT11 (https://www.parallax.com)	ATmega128L Marvell PXA271 TIMSP430 Arduino UNO TIMSP430 Cortex M3 LPC 17xx ARM 920T MSP430F2274 MSP430 MSP430G2553 MSP430F149 PIC24FJ64GB004 AT89C52 PIC18F452 PIC18F455 ATmega2560 AT86RF230 ATMega64L 8051 JN5148 ATmega1284P ARM9 ATmega328 MSP430F1611 PIC16F877A MSP430FR5739 AT89S52 STM 32/F4 ATmega1281 ATMEGA 16 PIC 18F452	ZigBee/IEEE 802.15.4 Bluetooth (IEEE 802.15.1) Bluetooth low energy (BLE) (IEEE 802.15.1) WiFi (IEEE 802.11) GPRS/3G/4G LoRa (IEEE 802.15.4g) SigFox (IEEE 802.15.4g)
